# Effects of Aspirin or Clopidogrel on Colorectal Cancer Chemoprevention in Patients with Type 2 Diabetes Mellitus

**DOI:** 10.3390/cancers11101468

**Published:** 2019-09-29

**Authors:** Yi-Chun Kuan, Kuang-Wei Huang, Cheng-Li Lin, Jiing-Chyuan Luo, Chia-Hung Kao

**Affiliations:** 1 Taipei Neuroscience Institute, Taipei Medical University, Taipei 11031, Taiwan; yckuang2@gmail.com; 2 Department of Neurology, Taipei Medical University-Shuang Ho Hospital, New Taipei City 23561, Taiwan; 3 Department of Neurology, School of Medicine, College of Medicine, Taipei Medical University, Taipei 11031, Taiwan; 4 Institute of Epidemiology and Preventive Medicine, College of Public Health, National Taiwan University, Taipei 10617, Taiwan; 5 Cochrane Taiwan, Taipei Medical University, Taipei 11031, Taiwan; 6 Center for Evidence-Based Health Care, Taipei Medical University—Shuang Ho Hospital, New Taipei City 23561, Taiwan; 7 Division of Gastroenterology, Department of Internal Medicine, Taipei Beitou Health Management Hospital, Taipei 11252, Taiwan; kwhuang3@gmail.com; 8 Management Office for Health Data, China Medical University Hospital, Taichung 40402, Taiwan; orangechengli@gmail.com; 9 College of Medicine, China Medical University, Taichung 40402, Taiwan; 10 Division of Gastroenterology, Department of Internal Medicine, Taipei Veterans General Hospital, Taipei 11217, Taiwan; jcluo@vghtpe.gov.tw; 11 Graduate Institute of Biomedical Sciences and School of Medicine, College of Medicine, China Medical University, Taichung 40402, Taiwan; 12 Department of Nuclear Medicine and PET Center, China Medical University Hospital, Taichung 40402, Taiwan; 13 Department of Bioinformatics and Medical Engineering, Asia University, Taichung 41454, Taiwan; 14 Center of Augmented Intelligence in Healthcare, China Medical University Hospital, Taichung 40402, Taiwan

**Keywords:** colorectal cancer, Type 2 diabetes mellitus, aspirin, clopidogrel

## Abstract

*Background:* The effect of clopidogrel, whose mechanism of action differs from that of aspirin, on CRC risk remains unknown. We investigated the effects of clopidogrel and aspirin, either as monotherapy or combined, on colorectal cancer (CRC) risk in patients with Type 2 diabetes mellitus (T2DM). *Methods:* We conducted a cohort study using Taiwan National Health Insurance Research Database. Four groups comprising 218,903 patients using aspirin monotherapy, 20,158 patients using clopidogrel monotherapy, 42,779 patients using dual antiplatelet therapy, and 281,840 nonuser matched controls were created using propensity score matching. Cox proportional hazards regression was used to evaluate the CRC risk during follow-up. *Results:* During the 13-year follow-up period, we found 9431 cases of CRC over 3,409,522 person-years. The overall incidence rates of CRC were 2.04, 3.45, 1.55, and 3.52 per 1000 person-years in the aspirin, clopidogrel, dual antiplatelet, and nonuser cohorts, respectively. The adjusted hazard ratios (aHRs) were 0.59 (95% confidence interval [CI], 0.56–0.61), 0.77 (95% CI, 0.68–0.87), and 0.37 (95% CI, 0.33–0.40) for the aspirin, clopidogrel, and dual antiplatelet cohorts, respectively. Dose- and duration-dependent chemopreventive effects were observed in the three cohorts.

## 1. Introduction

Colorectal cancer (CRC) is the third most common cancer and major leading cause of cancer-related deaths worldwide [1]. Over 1.8 million new CRC cases and 881,000 related deaths were estimated in 2018 [2]. The prevalence of CRC is expected to increase substantially in most developed countries because of population growth and aging. Consequently, the economic burden of CRC is considerable and is likely to increase over time [3]. The increasing prevalence of type 2 diabetes mellitus (T2DM) is also a major public health concern. The number of people with DM worldwide has doubled in the past three decades [4]. Epidemiological studies have suggested that T2DM is associated with an increased risk of several types of cancer, including CRC [5]. Furthermore, diabetes negatively affects overall survival in CRC [6]. Therefore, an effective prevention strategy may be critical for reducing the incidence and mortality rate of CRC in patients with T2DM.

The chemoprevention effect of aspirin against cancer incidence and mortality has been reported by a series of studies in the past few decades [7,8]. In particular, aspirin has demonstrated protective effects in a potentially dose-dependent manner for long-term CRC incidence and mortality as evidenced by a network meta-analysis [9]. The US Preventive Services Task Force (USPSTF) recommended the use of low-dose aspirin in the prevention of CRC in adults aged 50 to 59 adults aged 50 to 59 and 60 to 69 years, considering the 10-year cardiovascular risk, bleeding risk, and life expectancy. However, the populations examined in most studies are of limited number of T2DM patients [10,11,12]. Therefore, the role of aspirin in CRC chemoprevention in patients with T2DM remains unclear.

Clopidogrel, another antiplatelet agent, has a different mechanism of action from aspirin; clopidogrel irreversibly inhibits P2Y12 adenosine diphosphate-receptors. Recently, a few studies have reported promising results regarding the chemopreventive effect of clopidogrel in CRC, either alone or in combination with aspirin [13,14].

Whether aspirin and clopidogrel, either as monotherapy or combined, exhibit chemopreventive effects on CRC in patients with T2DM remains unknown. Therefore, we conducted a large population-based cohort study to investigate the CRC risk in patients with T2DM with and without aspirin or clopidogrel treatment.

## 2. Methods

### 2.1. Data Source

All data were acquired from the National Health Insurance Research Database (NHIRD), which was established by the National Health Research Institutes and insures over 99% of Taiwan residents. The database contains abundant health and medical information on the insurants, such as outpatient visits, inpatient visits, medication, operation treatment, and other medical care data. In the present study, we used the Longitudinal Cohort of Diabetes Patients, which includes patients who received new diagnoses of diabetes between 1999 and 2012 and whose medical records were collected between 1997 and 2013. We explored the association between aspirin or clopidogrel use and CRC (International Classification of Diseases, Ninth Revision, Clinical Modification [ICD-9-CM] codes: 153.x, 154.x). This study was approved by the Ethics Review Board of China Medical University (CMUH104-REC2-115-CR4). In addition, all diagnoses in the database were coded according to the ICD-9-CM.

### 2.2. Study Population

We included patients with T2DM with aspirin or clopidogrel prescription claims and over 28 cumulative defined daily doses (cDDDs) from 2001 to 2012 in the antiplatelet cohort. The date of the first prescription was selected as the index date. The main purpose of the current study was to determine whether the long-term, cumulative exposure to antiplatelet agents (aspirin or clopidogrel) exhibited any chemopreventive effects on CRC. Therefore, we considered patients without any exposure to aspirin and clopidogrel and those exposed to less than 28 cDDDs as part of the nonuser cohort. The antiplatelet user cohort was further divided into aspirin monotherapy, clopidogrel monotherapy, and dual antiplatelet therapy groups according to the prescription orders obtained during the follow-up period. We excluded patients younger than 20 years old, older than 85 years old, with missing data of sex, with preexisting CRC before the index date, or diagnosed as having CRC within a year after the index date. The antiplatelet user cohort and the nonuser cohort were matched using propensity score matching at a 1:1 ratio by age, sex, index year, comorbidities, Charlson comorbidity index scores (CCIs), adapted Diabetes Complications Severity Index (aDCSI), and medications.

### 2.3. Potential Confounders

The comorbidities and prescriptions of medications that could potentially confound the association between antiplatelet use and CRC risk were also identified. The baseline comorbidities were hypertension (ICD-9-CM codes 401–405), hyperlipidemia (ICD-9-CM codes 272.0–272.4), coronary artery disease (ICD-9-CM codes 410–414), stroke (ICD-9-CM codes 430–435), arrhythmia (ICD-9-CM code 427), chronic kidney disease (ICD-9-CM codes 585, 586), and chronic obstructive pulmonary disease (ICD-9-CM codes 491, 492, and 496). The prescriptions of medications included anti-DM drugs (metformin, sulfonylureas, alpha-glucosidase inhibitors, thiazolidinediones, and insulin), antihypertensive drugs (α-blockers, β-blockers, potassium-sparing diuretics, thiazides, loop diuretics, calcium channel blockers, angiotensin-converting enzyme inhibitors [ACEIs], and angiotensin II receptor blockers [ARBs]), statins, and nonsteroidal anti-inflammatory drugs (NSAIDs).

### 2.4. Main Outcome Measures

All patients were followed up from the index date until CRC diagnosis, death, withdrawal from the National Health Insurance (NHI) program, or 31 December 2013, whichever came first. The primary outcome of this study was the diagnosis of CRC (ICD-9-CM codes: 153.x, 154.x). We further stratified CRC into colon cancer (ICD-9-CM code: 153.x) and rectal cancer (ICD-9-CM code: 154.x). To test the exposure dose–effect and duration–effect relationships, we categorized the antiplatelet doses and use durations into four groups in each cohort (<28, 28 to 179, 180 to 359, and ≥360 cDDDs; <1, 1 to 2, 2 to 3, and ≥3 years).

### 2.5. Statistical Analysis

The statistical differences between the user and nonuser cohorts were determined through standardized mean difference (SMD). The SMD method has been suggested to measure the similarity of baseline characteristics in propensity-score-matched samples [15,16]. An SMD of less than 0.10 likely denotes a negligible imbalance between case patients and their matched controls [15]. Cox proportional hazards models were used to evaluate the hazard ratio (HR) and explore the association between aspirin or clopidogrel use and CRC. A multivariate Cox proportional hazards model was used to calculate the adjusted HRs (aHRs) after adjustment for age, sex, comorbidities, CCIs, aDCSI, and medications. An analysis of stratification by age, sex, CCIs, and aDCSI was performed to determine the association between aspirin or clopidogrel use and CRC among a specific population. Subhazard ratios (SHRs) were calculated using competing risk regression models considering the presence of the competing risk factor of death. The cumulative incidence of CRC was estimated using the Kaplan–Meier method, and a log-rank test was used to compare the incidence curves. All statistical analyses were performed using STATA/SE version 14.0 (STATA Corp., College Station, TX, USA). Statistical significance was determined through two-tailed tests (*p* < 0.05).

## 3. Results

### 3.1. Demographic Characteristics

**Figure 1:**
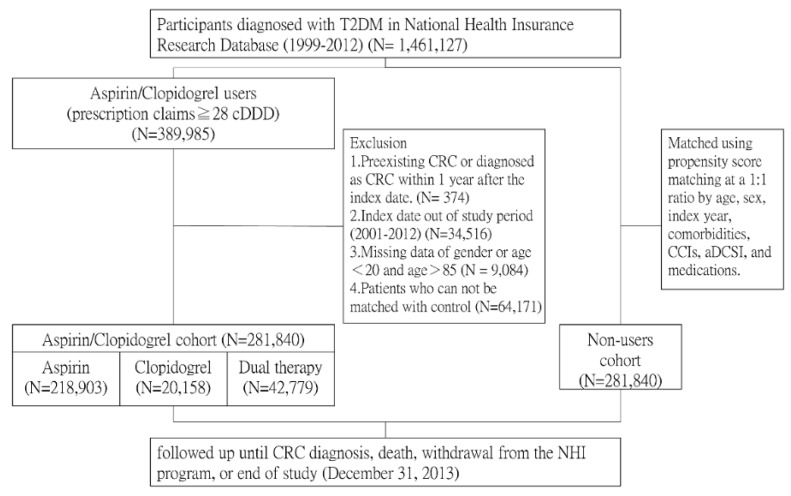
Flowchart of the current study.

In total, 281,840 antiplatelet users and 281,840 matched nonusers were included (Figure 1). Table 1 presents the demographic data, baseline characteristics, comorbidities, and medication use of the included patients. Clopidogrel users were generally older and had higher CCIs and aDCSI. After propensity score matching, the age, sex, comorbidities, and medication use did not differ between the antiplatelet user and nonuser cohorts (SMD ≤ 0.1).

### 3.2. Overall Incidence and Estimated HR of CRC

**Figure 2:**
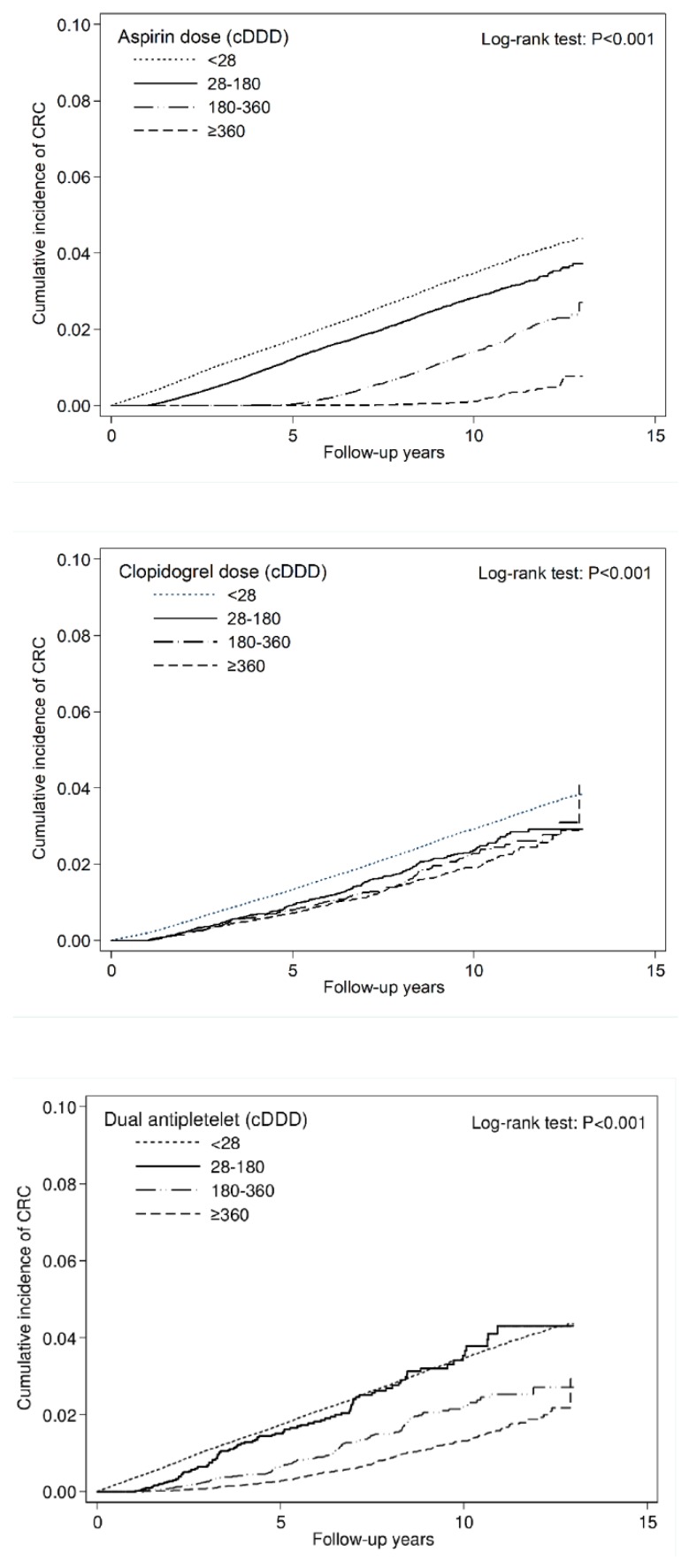
Cumulative incidence curve of CRC stratified by cumulative doses.

**Figure 3:**
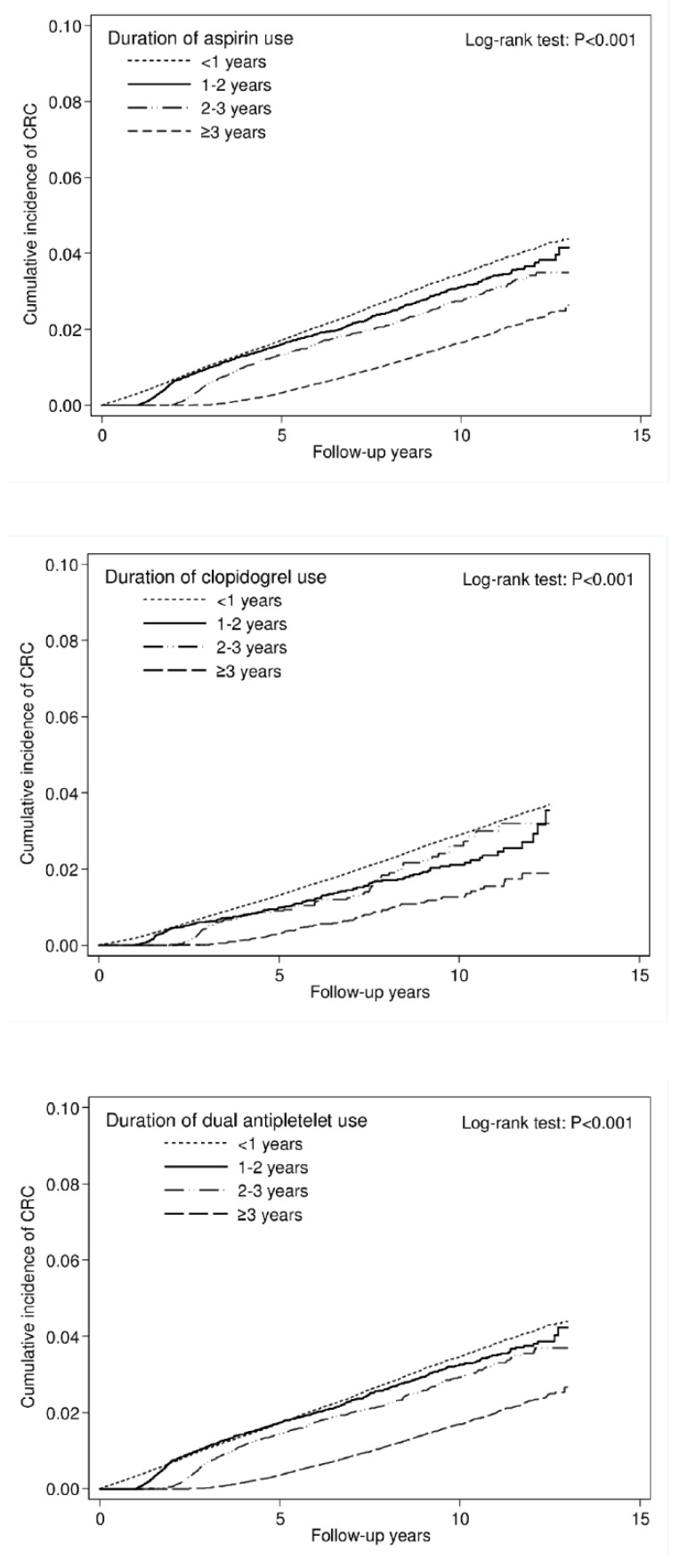
Cumulative incidence curve of CRC, stratified by duration.

During the 13-year follow-up period, we found 9431 cases of CRC over 3,409,522 person-years. The use of aspirin or clopidogrel significantly reduced the risk of CRC by nearly 45% (aHR = 0.55, 95% confidence interval [CI] = 0.53–0.58). The overall incidence rates of CRC were 2.04, 3.45, 1.55, and 3.52 per 1000 person-years in the aspirin monotherapy, clopidogrel monotherapy, dual antiplatelet therapy, and nonuser cohorts, respectively. Compared with nonusers, aspirin monotherapy, clopidogrel monotherapy, and dual antiplatelet therapy users had aHRs (95% CI) for CRC of 0.59 (0.56–0.61), 0.77 (0.68–0.87), and 0.37 (0.33–0.40), respectively. Moreover, we observed both dose- and duration-dependent effects. The risk of CRC was significantly decreased by an increased dose (measured in cDDD) of aspirin, clopidogrel, and dual antiplatelet therapy (aHRs were 0.73, 0.31, and 0.08 in the aspirin cohort; 0.63, 0.58, and 0.50 in the clopidogrel cohort; and 0.78, 0.44, and 0.28 in the dual antiplatelet cohort using 28–179, 180–359, and ≥360 cDDDs, respectively. *p* < 0.001 for trend). The same duration-dependent effect was also observed across the three cohorts (aHRs were 0.90, 0.71, and 0.38 in the aspirin cohort; 0.64, 0.63, and 0.31 in the clopidogrel cohort; 0.91, 0.84, and 0.34 in the dual antiplatelet cohort using the medications for 1–2, 2–3, and ≥3 years, respectively. *P* < 0.001 for trend) (Table 2). The significant dose- and duration-dependent decrease in cumulative incidence of CRC in aspirin, clopidogrel and dual antiplatelet cohorts is illustrated in Figure 2 and Figure 3.

### 3.3. Subgroup Analysis

Table 3 depicts the risk of CRC according to aspirin and clopidogrel exposure in several subgroups stratified by sex, age, CCIs, and aDCSI. In general, the chemopreventive effect of CRC was maintained across the three cohorts in various subgroups. However, the CRC risk was not significantly reduced when clopidogrel monotherapy was used in younger patients (<50 years old) and with an aDCSI <2.

### 3.4. Incidence and HR of CRC Stratified by Colon Cancer and Rectal Cancer

We further explored the association of aspirin or clopidogrel exposure with colon cancer (ICD-9-CM code: 153.x) and rectal cancer (ICD-9-CM code: 154.x). Antiplatelet use was beneficial to the reduction of the risk of both colon cancer (aHR = 0.56, 95% CI = 0.53–0.59) and rectal cancer (aHR = 0.54, 95% CI = 0.50–0.58). Aspirin monotherapy and dual antiplatelet therapy were significantly effective in both colon cancer and rectal cancer risk reduction. However, clopidogrel monotherapy was significantly effective only in colon cancer (aHR = 0.73, 95% CI = 0.63–0.85) but not in rectal cancer (aHR = 0.86, 95% CI = 0.69–1.07) risk reduction (Table 4).

### 3.5. Sensitivity Analysis after Considering the Competing Risk of Death

After considering death as a competing event, patients with aspirin or clopidogrel exposure still presented a lower risk of CRC than patients in the nonuser cohort (adjusted SHR [aSHR] = 0.58, 95% CI = 0.56–0.61). Compared with nonusers, aspirin monotherapy, clopidogrel monotherapy, and dual antiplatelet therapy users had aSHRs (95% CI) for CRC of 0.62 (0.59–0.65), 0.70 (0.62–0.79), and 0.40 (0.36–0.44), respectively. Dose-dependent effects across the three cohorts remained (Table 5).

## 4. Discussion

In the present study, aspirin monotherapy and clopidogrel monotherapy were associated with a decreased CRC risk (aspirin: aHR = 0.59, 95% CI = 0.56–0.61; clopidogrel: aHR = 0.77, 95% CI = 0.68–0.87). Dual antiplatelet therapy was associated with a lower CRC risk than aspirin monotherapy or clopidogrel monotherapy (aHR = 0.37, 95% CI = 0.33–0.40). Moreover, we observed dose- and duration-dependent preventive effects on the CRC risk across the three treatment cohorts. To our knowledge, our study is the first population-based cohort study to investigate the effect of aspirin and clopidogrel on the risk of CRC among real-world patients with T2DM in clinical practice.

Evidence from long-term follow-up randomized trials and observational studies supports that daily aspirin use reduces the incidence of and mortality rate induced by CRC [17,18]. In 2016, the USPSTF recommended the use of low-dose aspirin in the prevention of CRC in selected patients. However, most studies have included only a limited number of patients with T2DM [10,11,12]. Several epidemiological studies have suggested that T2DM was associated with an increased risk of CRC. However, whether aspirin plays the same protective role in this group has rarely been documented. The recent long-term observational follow-up of a randomized controlled trial in Japan evaluated the efficacy of aspirin on cancer chemoprevention mainly in patients with T2DM [19]. They concluded that low-dose aspirin did not reduce cancer incidence in most Japanese patients with T2DM apart from participants aged <65 years with CRC. However, these findings should be interpreted carefully considering that they were the results of a secondary analysis.

The current study provided several relevant clinical implications. Contrary to the available evidence from studies conducted on the general population, we focused on patients with T2DM who had a higher risk of CRC than the general population. Our results supported the current concept of aspirin use for CRC prevention. Furthermore, we noted that the benefit was dose- and duration-dependent, which was consistent with some reports [20,21]. The published study from Rodriguez-Miguel et al [14]. did not find relevant differences by aspirin doses (100 mg, adjusted OR 0.83(0.77–0.90); 125–300 mg, adjusted OR 0.79(0.71–0.89)). However, their results should be interpreted carefully due to some inconsistent results with a biologically plausible association (e.g., only current use but not recent or past use was associated with chemopreventive effect; short duration of use (1–3 years) was associated with lower risk than more than 3 years of use) and potential confounding exist (no significant association with CRC incidence in unadjusted analysis and only significant after adjusting for other risk factors). Moreover, these may have been attributed to their case-control study design, which was more prone to bias than the cohort study. In contrast to the study published from Rodriguez-Miguel et al., our current study adopted a cohort study design. The dose- and duration-dependent effect found in our current study may be supported by other analyses based on prospective cohort studies or clinical trials [8,20,21]. The protective effect appeared early over the treatment course, which may be explained by the preventive effect of aspirin in the adenoma–carcinoma sequence [22]. Two recent studies have demonstrated promising results among clopidogrel users, either as monotherapy or in combination with aspirin [13,14]. However, the lack of information on the potential confounding factors and the case-control study design could have yielded noncausal associations. To overcome these limitations, we conducted a large population-based cohort study considering most potential confounders such as comorbidities, disease severity index, and various medications. In this study, we demonstrated that clopidogrel use, both as monotherapy and in combination with aspirin, was associated with a reduction in CRC risk, which was in line with two recent epidemiologic studies from Leader et al. and Soriano et al. [13,23]. Furthermore, similarly to aspirin, a dose- and duration-dependent benefit was observed both in clopidogrel monotherapy and dual antiplatelet therapy.

Increasing evidence indicates that platelets play a key role in the development and progression of cancer [24]. Platelet activation results in inflammatory reaction through the release of proinflammatory mediators. Furthermore, activated platelets may promote cancer development through chronic inflammation and the secretion of a variety of cytokines and growth factors [25]. The anticancer mechanisms of aspirin and clopidogrel may be complex and dissimilar. Clopidogrel inhibits platelet function through the blockage of P2Y12 adenosine diphosphate-receptor. Apart from the inactivation of platelet property through COX-1, aspirin exerts other anticancer effects through several interconnected mechanisms, including prostaglandin synthesis and catabolism in epithelial cells, inhibition of WNT–*β*-catenin signaling, and modulation of the host immune response [26]. These may explain the difference between the chemopreventive effects of aspirin and clopidogrel. In addition, aspirin has been reported to improve glucose tolerance and insulin sensitivity [27,28]. Insulin resistance may also play a role in colon carcinogenesis [29]. Clopidogrel has also been reported to benefit glycemic indices and reduced oxidative stress in T2DM patients [30]. These may explain the possible additive effect of dual antiplatelet therapy in T2DM patients observed in the current study but not in published study from Rodriguez-Miguel et al. [14] We also noted that clopidogrel monotherapy did not exhibit effective chemopreventive effects on rectal cancer. Distal colon and rectal cancers tend to express higher levels of COX-2 than proximal colon cancers [31]. The ability of aspirin to irreversibly inhibit COX-2 has often been considered to be central in its chemopreventive mechanisms. This may explain why aspirin but not clopidogrel had prevention effects in rectal cancer in the current study.

This study presents several advantages that increase the validity of our results. First, the population-based cohort study design, which involved using data from the NHIRD, yielded relatively large sample sizes and made the findings generalizable. Secondly, we used a propensity score matching approach to adjust for several confounding variables such as age, sex, underlying comorbidities, aDCSI, and medications, which may have reduced any potential selection bias. Thirdly we also evaluated the effects of exposure duration and cumulative dosage. Fourthly, we performed a sensitivity analysis by considering the competing risk of death.

However, the present study still has some limitations that should be addressed. First, this was a retrospective cohort study, and the study population was selected from a claims-based dataset. Evidence derived from a retrospective cohort study is generally of a lower methodological quality than that obtained from randomized trials. However, a randomized controlled trial is usually difficult to evaluate and to follow-up for long-time outcomes, such as cancer development. Secondly, the NHIRD provides inadequate detailed personal information regarding genetic factors, body mass index, lifestyle, dietary habits, smoking or alcohol intake, which are possible confounding factors.

In conclusion, this study demonstrates that clopidogrel monotherapy, as well as aspirin monotherapy, are associated with dose- and duration-dependent protective effects on CRC in patients with T2DM. The combination of aspirin and clopidogrel was associated with additional benefits. The findings of the current study, along with those of recently published studies [13,14,23], support the hypothesis of a platelet-mediated mechanism in CRC and a possible antiplatelet-based chemoprevention strategy.

## 5. Conclusions

Both aspirin and clopidogrel monotherapies reduced the CRC risk in patients with T2DM in a dose- and duration-dependent manner. The combination of aspirin and clopidogrel was associated with additional benefits.

## Figures and Tables

**Table 1:** Baseline distribution of the demographics and clinical comorbidities in the study cohorts.

Variables	Nonusers N (%)	Users			Standardized Mean Difference §
		Aspirin Monotherapy N (%)	Clopidogrel Monotherapy N (%)	Dual Antiplatelet N (%)	
	*N* = 281,840	*N* = 218,903	*N* = 20,158	*N* = 42,779	
**Sex**					0.016
Female	131,113 (46.5)	105,795 (48.3)	8403 (41.7)	14,719 (34.4)	
Male	150,727 (53.5)	113,108 (51.7)	11,755 (58.3)	28,060 (65.6)	
**Age, Years**					
<50	47,336 (16.8)	37,300 (17.0)	1725 (8.6)	6196 (14.5)	0.020
50–59	77,979 (27.7)	66,656 (30.5)	4163 (20.7)	11,854 (27.7)	0.037
60–69	76,223 (27.0)	62,570 (28.6)	5465 (27.1)	12,880 (30.1)	0.037
70–85	80,302 (28.5)	52,377 (23.9)	8805 (43.7)	11,849 (27.7)	0.058
Mean (SD)	61.9 (12.0)	61.0 (11.3)	66.6 (11.3)	62.3 (11.1)	0.027
**Comorbidities**					
Hypertension	229,050 (81.3)	171,934 (78.5)	16,805 (83.4)	33,324 (77.9)	0.062
Hyperlipidemia	166,209 (59.0)	127,098 (58.1)	11,427 (56.7)	24,046 (56.2)	0.026
CAD	106,197 (37.7)	79,578 (36.4)	11,205 (55.6)	20,904 (48.9)	0.040
Stroke	40,658 (14.4)	31,205 (14.3)	7154 (35.5)	6865 (16.1)	0.045
Arrhythmia	47,949 (17.0)	36,154 (16.5)	5294 (26.3)	7761 (18.1)	0.012
CKD	14,252 (5.1)	8464 (3.9)	2896 (14.4)	2643 (6.2)	0.004
COPD	88,872 (31.5)	65,698 (30.0)	8359 (41.5)	14,000 (32.7)	0.006
**CCIs**					
0	176,684 (62.7)	140,110 (64.0)	4612 (22.9)	18,781 (43.9)	0.096
1	49,155 (17.4)	41,522 (19.0)	4537 (22.5)	11,885 (27.8)	0.080
2	25,954 (9.2)	20,886 (9.5)	4114 (20.4)	6766 (15.8)	0.068
≥3	30,047 (10.7)	16,385 (7.5)	6895 (34.2)	5347 (12.5)	0.017
**aDCSI**					
0	73,470 (26.1)	53,935 (24.6)	1289 (6.4)	8056 (18.8)	0.084
1	51,599 (18.3)	42,006 (19.2)	1527 (7.6)	6707 (15.7)	0.013
≥2	156,771 (55.6)	122,962 (56.2)	17,342 (86.0)	28,016 (65.5)	0.083
**Medications**					
**Anti-DM drugs**					
Metformin	130,138 (46.2)	99,753 (45.6)	8876 (44.0)	17,812 (41.6)	0.026
Sulfonylureas	134,766 (47.8)	101,823 (46.5)	9032 (44.8)	20,271 (47.4)	0.026
AGI	24,870 (8.8)	17,103 (7.8)	2506 (12.4)	3200 (7.5)	0.026
Thiazolidinediones	23,513 (8.3)	15,933 (7.3)	2238 (11.1)	2953 (6.9)	0.031
Insulin	41,127 (14.6)	26,729 (12.2)	6188 (30.7)	6736 (15.8)	0.015
**Anti-HTN drugs**					
*α*-Blockers	70,952 (25.2)	51,343 (23.5)	7832 (38.9)	12,548 (29.3)	0.006
*β*-Blockers	198,753 (70.5)	149,804 (68.4)	156,68 (77.7)	30,142 (70.5)	0.024
PSD	21,794 (7.7)	14,969 (6.8)	3324 (16.5)	3553 (8.3)	0.001
Thiazides	78,801 (28.0)	60,352 (27.6)	6964 (34.6)	12,008 (28.1)	0.004
Loop diuretics	95,472 (33.9)	68,950 (31.5)	10,658 (52.9)	15,516 (36.3)	0.003
CCBs	207,337 (73.6)	155,461 (71.0)	16,236 (80.5)	30,879 (72.2)	0.038
ACEIs	171,116 (60.7)	128,789 (58.8)	13,856 (68.7)	26,076 (61.0)	0.017
ARBs	88,480 (31.4)	68,309 (31.2)	9568 (47.5)	14,261 (33.3)	0.028
**Statins**	106,396 (37.8)	79,589 (36.4)	9453 (46.9)	16,766 (39.2)	0.004
**NSAIDs**	258,544 (91.7)	201,541 (92.1)	18,242 (90.5)	38,551 (90.1)	0.003

Abbreviations: SD: standard deviation; CAD: coronary artery disease; CKD: chronic kidney disease; COPD: chronic obstructive pulmonary disease; CCI: Charlson Comorbidity Index; aDCSI: adapted Diabetes Complications Severity Index; DM: diabetes mellitus; AGI: alpha-glucosidase inhibitors; HTN: hypertension; PSD: potassium-sparing diuretics; CCBs: calcium channel blockers; ACEIs: angiotensin-converting-enzyme inhibitors; ARBs: angiotensin-receptor blockers; NSAIDs: nonsteroidal anti-inflammatory drugs. § A standardized mean difference ≤ 0.1 indicates a negligible difference between the nonuser and user cohorts.

**Table 2:** Incidence rate and hazard ratio of colorectal cancer (CRC) in patients with Type 2 diabetes mellitus (T2DM) using or not aspirin and clopidogrel.

Variables	No. non-CRC (%)	No. CRC (%)	Person-Years	IR^#^	Crude HR (95% CI)	*p* value	Adjusted HR (95% CI) ^†^	*p* value
Aspirin or Clopidogrel								
Nonusers	276082 (98.0)	5758 (2.0)	1635323	3.52	Reference		Reference	
Users	278257 (98.7)	3583 (1.3)	1774199	2.02	0.57 (0.55–0.59)	<0.001	0.55 (0.53–0.58)	<0.001
Aspirin or Clopidogrel								
Nonusers	276082 (98.0)	5758 (2.0)	1635323	3.52	Reference		Reference	
Aspirin Monotherapy	216058 (98.7)	2845 (1.3)	1393473	2.04	0.58 (0.55–0.60)	<0.001	0.59 (0.56–0.61)	<0.001
Clopidogrel Monotherapy	19888 (98.7)	270 (1.3)	78279.76	3.45	1.03 (0.91–1.17)	0.609	0.77 (0.68–0.87)	<0.001
Dual Antiplatelet Therapy	42311 (98.9)	468 (1.1)	302446	1.55	0.43 (0.39–0.47)	<0.001	0.37 (0.33–0.40)	<0.001
Dose of Aspirin Used								
<28 cDDDs	295970 (98.0)	6028 (2.0)	1713603	3.52	Reference		Reference	
28–179 cDDDs	185267 (98.6)	2661 (1.4)	1053000	2.53	0.72 (0.69–0.76)	<0.001	0.73 (0.69–0.76)	<0.001
180–359 cDDDs	64802 (99.1)	621 (1.0)	549969.8	1.13	0.31 (0.28–0.33)	<0.001	0.31 (0.28–0.33)	<0.001
≥360 cDDDs	8300 (99.6)	31 (0.4)	92948.86	0.33	0.09 (0.06–0.12)	<0.001	0.08 (0.06–0.12)	<0.001
*p* for Trend						<0.001		<0.001
Dose of Clopidogrel Used								
<28 cDDDs	492140 (98.3)	8603 (1.7)	3028796	2.84	Reference		Reference	
28–179 cDDDs	22691 (98.8)	280 (1.2)	130777	2.14	0.75 (0.67–0.85)	<0.001	0.63 (0.56–0.71)	<0.001
180–359 cDDDs	13075 (98.9)	148 (1.1)	76987.56	1.92	0.68 (0.58–0.80)	<0.001	0.58 (0.49–0.68)	<0.001
≥360 cDDDs	26433 (98.8)	310 (1.2)	172961.2	1.79	0.63 (0.56–0.70)	<0.001	0.50 (0.44–0.56)	<0.001
*P* for trend						<0.001		<0.001
Dose of Dual Antiplatelet Used								
<28 cDDDs	276082 (98.0)	5758 (2.0)	1635323	3.52	Reference		Reference	
28–179 cDDDs	5294 (98.2)	100 (1.9)	30816.84	3.24	0.92 (0.76–1.12)	0.421	0.78 (0.64–0.95)	0.014
180–359 cDDDs	11490 (98.9)	133 (1.1)	73431.77	1.81	0.51 (0.43–0.61)	<0.001	0.44 (0.37–0.53)	<0.001
≥360 cDDDs	25527 (99.1)	235 (0.9)	198197.4	1.19	0.33 (0.29–0.38)	<0.001	0.28 (0.25–0.32)	<0.001
*P* for trend						<0.001		<0.001
Duration of Aspirin Use, Years								
<1	313173 (98.0)	6305 (2.0)	1807507	3.49	Reference		Reference	
1–2	64177 (98.5)	999 (1.5)	326200.2	3.06	0.89 (0.83–0.95)	0.001	0.90 (0.84–0.96)	0.002
2–3	44925 (98.6)	620 (1.4)	249687.8	2.48	0.72 (0.66–0.78)	<0.001	0.71 (0.65–0.77)	<0.001
≥3	132064 (98.9)	1417 (1.1)	1026127	1.38	0.38 (0.36–0.41)	<0.001	0.38 (0.36–0.40)	<0.001
*P* for trend						<0.001		<0.001
Duration of Clopidogrel Use, Years								
<1	527837 (98.3)	9039 (1.7)	3236326	2.79	Reference		Reference	
1–2	12145 (98.8)	152 (1.2)	71455.8	2.13	0.76 (0.65–0.90)	0.001	0.64 (0.54–0.75)	<0.001
2v3	5409 (98.7)	73 (1.3)	33470.92	2.18	0.78 (0.62–0.98)	0.036	0.63 (0.50–0.79)	<0.001
≥3	8948 (99.2)	77 (0.9)	68269.17	1.13	0.40 (0.32–0.50)	<0.001	0.31 (0.25–0.39)	<0.001
*p* for trend						<0.001		<0.001
Duration of Dual Antiplatelet Use, Years								
<1	299519 (98.0)	6114 (2.0)	1744497	3.50	Reference		Reference	
1–2	63439 (98.4)	1009 (1.6)	309113.5	3.26	0.95 (0.89–1.02)	0.155	0.96 (0.89–1.02)	0.194
2–3	46385 (98.6)	651 (1.4)	246389.1	2.64	0.77 (0.71–0.83)	<0.001	0.75 (0.69–0.81)	<0.001
≥3	144996 (98.9)	1567 (1.1)	1109523	1.41	0.39 (0.37–0.41)	<0.001	0.38 (0.36–0.40)	<0.001
*p* for trend						<0.001		<0.001

IR^#^, incidence rate (per 1,000 person-years); HR, hazard ratio. ^†^ Adjusted sex, age, comorbidities, CCIs, aDCSI, and medications listed in Table 1.

**Table 3:** Hazard ratio of CRC in patients with T2DM using aspirin and clopidogrel or not, stratified by sex, age, CCIs, and aDCSI.

Variables	Nonusers	Aspirin Monotherapy		Clopidogrel Monotherapy		Dual Antiplatelet Therapy	
		Adjusted HR (95% CI) ^†^	*p* value	Adjusted HR (95% CI) ^†^	*p* value	Adjusted HR (95% CI) ^†^	*p* value
Sex							
Female	Reference	0.57 (0.53–0.61)	<0.001	0.77 (0.63–0.94)	0.01	0.39 (0.33–0.45)	<0.001
Male	Reference	0.60 (0.56–0.64)	<0.001	0.76 (0.65–0.90)	0.001	0.36 (0.32–0.40)	<0.001
Age							
<50	Reference	0.58 (0.48–0.69)	<0.001	0.88 (0.45–1.73)	0.714	0.47 (0.32–0.69)	<0.001
50–59	Reference	0.59 (0.54–0.65)	<0.001	0.58 (0.40–0.86)	0.006	0.43 (0.35–0.52)	<0.001
60–69	Reference	0.54 (0.50–0.58)	<0.001	0.70 (0.55–0.88)	0.002	0.32 (0.27–0.37)	<0.001
70–85	Reference	0.63 (0.59–0.68)	<0.001	0.85 (0.72–1.00)	0.048	0.37 (0.31–0.42)	<0.001
CCIs							
0	Reference	0.60 (0.57–0.64)	<0.001	0.90 (0.71–1.14)	0.361	0.35 (0.31–0.41)	<0.001
1	Reference	0.56 (0.51–0.62)	<0.001	0.66 (0.51–0.86)	0.002	0.39 (0.32–0.46)	<0.001
2	Reference	0.56 (0.49–0.64)	<0.001	0.69 (0.52–0.91)	0.008	0.36 (0.28–0.45)	<0.001
≥3	Reference	0.60 (0.52–0.70)	<0.001	0.80 (0.64–1.01)	0.057	0.34 (0.26–0.45)	<0.001
aDCSI							
0	Reference	0.62 (0.57–0.68)	<0.001	0.97 (0.62–1.53)	0.898	0.31 (0.24–0.40)	<0.001
1	Reference	0.59 (0.53–0.66)	<0.001	0.94 (0.63–1.42)	0.78	0.35 (0.28–0.45)	<0.001
≥2	Reference	0.57 (0.54–0.61)	<0.001	0.73 (0.64–0.84)	<0.001	0.38 (0.34–0.43)	<0.001

^†^ Adjusted sex, age, comorbidities, CCIs, aDCSI, and medications listed in Table 1.

**Table 4:** Incidence rate and hazard ratio of colon cancer and rectal cancer in patients with T2DM using aspirin and clopidogrel or not.

Variables	No. non-CRC (%)	No. CRC (%)	Person-Years	IR^#^	Crude HR (95% CI)	*p* value	Adjusted HR (95% CI) ^†^	*p* value
Colon Cancer (ICD-9-CM code: 153.x) Aspirin or Clopidogrel Use								
Nonusers	277,711 (98.5)	4129 (1.5)	1,635,323	2.52	Reference		Reference	
Users	279,244 (99.1)	2596 (0.9)	1,774,199	1.46	0.57 (0.55–0.60)	<0.001	0.56 (0.53–0.59)	<0.001
Aspirin or Clopidogrel Use								
Nonusers	277711 (98.5)	4129 (1.5)	1635323	2.52	Reference		Reference	
Aspirin Monotherapy	216844 (99.1)	2059 (0.9)	1393473	1.48	0.58 (0.55–0.61)	<0.001	0.59 (0.56–0.63)	<0.001
Clopidogrel Monotherapy	19972 (99.1)	186 (0.9)	78279.76	2.38	1.00 (0.86–1.16)	0.98	0.73 (0.63–0.85)	<0.001
Dual Antiplatelet Therapy	42428 (99.2)	351 (0.8)	302446	1.16	0.45 (0.40–0.50)	<0.001	0.38 (0.34–0.43)	<0.001
Rectal Cancer (ICD-9-CM code: 154.x) Aspirin or Clopidogrel Use								
Nonusers	280166 (99.4)	1674 (0.6)	1635323	1.02	Reference		Reference	
Users	280829 (99.6)	1011 (0.4)	1774199	0.57	0.55 (0.51–0.60)	<0.001	0.54 (0.50–0.58)	<0.001
Aspirin or Clopidogrel Use								
Nonusers	280166 (99.4)	1674 (0.6)	1635323	1.02	Reference		Reference	
Aspirin Monotherapy	218100 (99.6)	803 (0.4)	1393473	0.58	0.56 (0.51–0.61)	<0.001	0.57 (0.52–0.62)	<0.001
Clopidogrel Monotherapy	20072 (99.6)	86 (0.4)	78279.76	1.10	1.11 (0.90–1.39)	0.326	0.86 (0.69–1.07)	0.167
Dual Antiplatelet Therapy	42657 (99.7)	122 (0.3)	302446	0.40	0.39 (0.32–0.47)	<0.001	0.33 (0.27–0.40)	<0.001

^†^ Adjusted sex, age, comorbidities, CCIs, aDCSIm and medications listed in Table 1.

**Table 5:** Incidence rate and subhazard ratio of CRC in patients using aspirin and clopidogrel or not, using competing risks regression models.

Variables	Crude SHR (95% CI)	*p* value	Adjusted SHR (95% CI) ^†^	*p* value
Aspirin or Clopidogrel Use				
Nonusers	Reference		Reference	
Users	0.59 (0.56–0.61)	<0.001	0.58 (0.56–0.61)	<0.001
Aspirin or Clopidogrel Use				
Nonusers	Reference		Reference	
Aspirin Monotherapy	0.60 (0.57–0.63)	<0.001	0.62 (0.59–0.65)	<0.001
Clopidogrel Monotherapy	0.87 (0.77–0.98)	0.020	0.70 (0.62–0.79)	<0.001
Dual Antiplatelet Therapy	0.45 (0.41–0.49)	<0.001	0.40 (0.36–0.44)	<0.001
Dose of Aspirin Used				
<28 cDDDs	Reference		Reference	
28–179 cDDDs	0.74 (0.71–0.78)	<0.001	0.75 (0.72–0.79)	<0.001
180–359 cDDDs	0.33 (0.31–0.36)	<0.001	0.34 (0.31–0.37)	<0.001
≥360 cDDDs	0.10 (0.07–0.14)	<0.001	0.10 (0.07–0.14)	<0.001
*p* for trend		<0.001		<0.001
Dose of Clopidogrel Used				
<28 cDDDs	Reference		Reference	
28–179 cDDDs	0.70 (0.63–0.79)	<0.001	0.61 (0.54–0.69)	<0.001
180–359 cDDDs	0.66 (0.56–0.78)	<0.001	0.59 (0.50–0.69)	<0.001
≥360 cDDDs	0.63 (0.56–0.71)	<0.001	0.53 (0.47–0.59)	<0.001
*p* for trend		<0.001		<0.001
Dose of Dual Antiplatelet Used				
<28 cDDDs	Reference		Reference	
28–179 cDDDs	0.90 (0.74–1.09)	0.272	0.79 (0.65–0.97)	0.023
180–359 cDDDs	0.52 (0.44–0.62)	<0.001	0.47 (0.40–0.56)	<0.001
≥360 cDDDs	0.35 (0.31–0.40)	<0.001	0.31 (0.27–0.36)	<0.001
*p* for trend		<0.001		<0.001
Duration of Aspirin Use, Years				
<1				
1–2	0.89 (0.83–0.95)	<0.001	0.90 (0.85–0.97)	0.0034
2–3	0.73 (0.68–0.80)	<0.001	0.74 (0.68–0.80)	<0.001
≥3	0.41 (0.39–0.44)	<0.001	0.42 (0.39–0.44)	<0.001
*p* for trend		<0.001		<0.001
Duration of Clopidogrel Use, Years				
<1				
1–2	0.75 (0.64–0.88)	<0.001	0.66 (0.56–0.77)	<0.001
2–3	0.77 (0.62–0.97)	0.0292	0.66 (0.52–0.83)	<0.001
≥3	0.41 (0.33–0.51)	<0.001	0.34 (0.27–0.43)	<0.001
*p* for trend		<0.001		<0.001
Duration of Dual Antiplatelet Use, Years				
<1				
1–2	0.94 (0.88–1.00)	0.0635	0.95 (0.88–1.01)	0.1008
2–3	0.77 (0.71–0.84)	<0.001	0.77 (0.71–0.83)	<0.001
≥3	0.42 (0.40–0.44)	<0.001	0.41 (0.39–0.44)	<0.001
*p* for trend		<0.001		<0.001

IR^#^, incidence rate (per 1,000 person-years); SHR, subhazard ratio. ^†^ Adjusted sex, age, comorbidities, CCIs, aDCSI, and medications listed in Table 1.
